# Alendronate-Grafted Nanoemulsions for Bone-Targeted Vincristine Delivery: Preliminary Studies on Cell and Animal Models

**DOI:** 10.3390/biom14020238

**Published:** 2024-02-18

**Authors:** Ian Stoppa, Chiara Dianzani, Nausicaa Clemente, Annalisa Bozza, Valentina Bordano, Sara Garelli, Luigi Cangemi, Umberto Dianzani, Luigi Battaglia

**Affiliations:** 1 Department of Health Sciences, Interdisciplinary Research Center of Autoimmune Diseases (IRCAD), University of Eastern Piedmont (UPO), via Solaroli 17, 28100 Novara, Italy; ian.stoppa@uniupo.it (I.S.); nausicaa.clemente@med.uniupo.it (N.C.); umberto.dianzani@med.uniupo.it (U.D.); 2 Department of Drug Science and Technology, University of Turin, via Pietro Giuria 9, 10125 Turin, Italy; chiara.dianzani@unito.it (C.D.); annalisa.bozza@unito.it (A.B.); valentina.bordano@unito.it (V.B.); saragarelli.sg@gmail.com (S.G.); luigi.cangemi@unito.it (L.C.); 3 Nanostructured Interfaces and Surfaces (NIS) Interdepartmental Centre, University of Turin, 10124 Turin, Italy

**Keywords:** nanoemulsion, alendronate, vincristine

## Abstract

Bone is a site of distant metastases, which are a common cause of morbidity and mortality with a high socio-economic impact, for many malignant tumours. In order to engineer pharmacological therapies that are suitable for this debilitating disease, this experimental work presents injectable lipid nanoemulsions, which are endowed with a long history of safe clinical usage in parenteral nutrition, their loading with vincristine and their grafting with alendronate, with a dual purpose: merging the anticancer activity of bisphosphonates and vincristine, and enhancing bone-targeted delivery. In cell studies, alendronate synergised with the anti-migration activity of vincristine, which is important as migration plays a key role in the metastatisation process. In preliminary animal studies, carried out thanks to IVIS technology, alendronate conjugation enhanced the bone targeting of fluorescently labelled nanoemulsions. These encouraging results will drive further studies on suitable animal models of the disease.

## 1. Introduction

Bone is a potential target for distant metastases in a variety of malignant tumours, which are also commonly found *post mortem*. Indeed, bone is the third most frequent site of metastasis after the lungs and liver. Prostate and breast cancer are responsible for the majority of skeletal metastases (up to 70%), followed by thyroid, lung, bladder and kidney neoplasias, myeloma and melanoma [1]. Bone metastases are a major cause of morbidity and are characterised by severe pain, impaired mobility, pathologic fractures, spinal cord compression, bone marrow aplasia and hypercalcemia. They are therefore a common cause of morbidity and mortality, with high socio-economic impact [2].

Bisphosphonates are analogues of pyrophosphate, a natural inhibitor of bone demineralisation. They bind avidly to exposed bone mineral around resorbing osteoclasts, where they are internalised, causing disruption to several biochemical processes involved in bone resorption, and ultimately leading to apoptotic cell death. In oncology, bisphosphonates are an innovative therapy for bone metastasis, and have an effect that is independent of the nature of the primary tumour and the type of lesion; this lesion can either be sclerotic (characterised by deposition of new bone), or lytic (characterised by destruction of normal bone). Bisphosphonates are able to rapidly restore normocalcemia and exert their antitumour effects by interrupting the increased osteolysis that is associated with increased tumour growth, thus preserving bone health and delaying bone lesion progression [1]. Moreover, some bisphosphonates (in particular zoledronic acid) can have direct cytotoxic effects on cancer cells and on the innate immune mechanisms, thus contributing to the antitumor effect [3].

Vincristine, a microtubule polymerisation inhibitor, is a broad-spectrum cytotoxic agent that is widely employed in oncology [4]. It has also been reported to act as an efficacious treatment against skeletal metastases from medulloblastoma [5]. It is worth noting that, owing to its mechanism of action, it is a well-known inhibitor of cell migration, which underlies the metastatisation processes from the primary tumour to the bones. Clinically, it is administered in combination with other drugs to reduce adverse drug effects, especially bone marrow inhibition, that may be caused by mono-therapy. However, its low cancer affinity means that off-target biodistribution can lead to considerable side effects, including irreversible neurotoxicity, which are currently a relevant limitation to its usage [4].

Nanotechnologies are versatile tools in this context as they can be used in the repurposing of existing cancer chemotherapeutics with an innovative delivery approach, which can improve pharmacokinetics and specific biodistribution to the target site, thus improving the drug’s therapeutic index [6]. In the case of metastatic bone tumours, bisphosphonates’ aforementioned properties can be exploited in achieving bone-targeted and nanocarrier-mediated vincristine delivery. Indeed, nanocarriers can be suitably engineered with bisphosphonate grafted to the surface and vincristine loaded into the core, with a dual purpose: (1) merging the anticancer activity of bisphosphonates and vincristine in the same vehicle, and (2) enhancing targeted drug delivery to bone, leading to increased efficacy and a reduction in side effects.

Their long history of safe clinical usage in parenteral nutrition [7], and in drug delivery (anaesthetics, diazepam, corticosteroids and anti-inflammatory drugs) [8], means that injectable lipid nanoemulsions have recently been proposed for the targeted delivery of combined chemotherapies to treat various cancers [9,10,11,12]. In this experimental work, the Intralipid^®^ 10% (IL) nanoemulsion was loaded with vincristine and surface-grafted with alendronate sodium (ALD), a first-generation bisphosphonate, via thiol–maleimide chemistry. Engineered nanoemulsions were tested on available osteosarcoma cell models, and fluorescently labelled formulations were then tested *ex vivo* on isolated mouse scapulae and *in vivo* on healthy mice for preliminary biodistribution studies, with the help of IVIS (IVIS Spectrum, PerkinElmer, Waltham, MA, USA) technology.

## 2. Materials and Methods

### 2.1. Chemicals

3-(4,5-dimethyl thiazol-2-yl)-2,5-diphenyltetrazolium bromide (MTT), 6-coumarin (6-cou), acetonitrile, alendronate sodium (ALD), chloroform, crystal violet, dimethylsulfoxide (DMSO), ethanol, methanol, Nile Red (NR), sodium dodecyl sulfate (SDS), stearylamine (ST) and vincristine sulfate were purchased from Sigma-Aldrich (St. Louis, MO, USA). Eriochrome black T (NET), the Millipore MilliQ system (for deionised water), sodium dioctylsulfosuccinate (AOT) and tromethamol (TRIS) were purchased from Merck (Darmstadt, Germany). 2-[2-[2-chloro-3-[(1,3-dihydro-3,3-dimethyl-1-propyl-2H-indol-2-ylidene)ethylidene]-1-cyclohexen-1-yl]ethenyl]-3,3-dimethyl-1-propylindolium (IR780) iodide and 3-maleimidobenzoic acid N-hydroxysuccinimide ester (MBS) were purchased from Alfa Aesar (Haverhill, MA, USA). Calcium bromide (CaBr_2_), calcium phosphate (Ca_3_(PO_4_)_2_), ethylenediaminetetraacetic acid sodium salt (EDTA) and sodium hydroxide (NaOH) were purchased from Carlo Erba (Cornaredo, Italy). Sodium monohydrogen and dihydrogen phosphate were purchased from A.C.E.F. (Fiorenzuola D’Arda, Italy). IL (10% Intralipid^®^) was purchased from BBraun. All other chemicals were of analytical grade and used without any further purification.

### 2.2. Cells

U2OS and HOS human osteosarcoma cells were purchased from the American Type Culture Collection (ATCC; Manassas, VA, USA). U2OS cells were cultured in McCoy’s 5A Medium and HOS cells in EMEM (Eagles’s Minimum Essential Medium) (Sigma-Aldrich, St. Louis, MO, USA). All culture media were supplemented with 10% Foetal Calf Serum (FCS; PAA Laboratories, Pasching, Austria), penicillin/streptomycin (100 units/mL) and L-glutamine (2 mmol/L) (both from Sigma-Aldrich, St. Louis, MO, USA). HOS were supplemented with 1% non-essential amino acids (Sigma-Aldrich, St. Louis, MO, USA). Cells were cultured in a 5% CO_2_, 37 °C incubator.

### 2.3. Animals

BALB/c mice were obtained from The Jackson Laboratory (Bar Harbor, ME, USA) and were then bred under pathogen-free conditions in the animal facility at the University of Eastern Piedmont, Department of Health Sciences (Authorization No. 61/2005-A—6 May 2005, issued by the General Directorate of Veterinary and Food Health—Italian Ministry of Health). Experiments were performed using male, 6-to-9-week-old BALB/c mice that were treated in accordance with the University Ethical Committee and European guidelines (experimental protocol authorization No. 241/2022-PR, released on the 15-04-2022 by the Italian Ministry of Health for protocol No. DB064.76).

### 2.4. Methods

#### 2.4.1. Synthesis/Modification of Compounds

##### Synthesis of ST-MBS

The lipophilic maleimide-linker ST-MBS was synthesised following a literature method [13].

##### IR780-SDS Ion-Pair Formation

Given that IR780 iodide hampers IL stability, iodide was substituted with the SDS counterion using the following procedure: 5 mg (7 μmol) of IR-780 iodide was dissolved in 1.5 mL methanol and subsequently diluted with 1.5 mL water. Then, 2 mg (7 μmol) of SDS was added to this solution. After vortexing, a precipitate formed (IR-780/SDS ion pair), and this was isolated via centrifugation at 2400× *g* (Rotofix 32 centrifuge, Hettich, Tuttingen, Germany) for 5 min, and dried under nitrogen steam. The IR780-SDS molar ratio was assessed using UV–VIS spectrophotometric analysis (Lambda 2 UV–vis spectrophotometer, Perkin Elmer, Waltham, MA, USA) at λ = 705 nm.

#### 2.4.2. Formulation Engineering

##### Formulation of Vincristine–AOT-Loaded Nanoemulsions

Vincristine sulphate was pre-dissolved in DMSO (20 mg/mL) and AOT was pre-dissolved in water (4.5 mg/mL). A total of 5 μL of the vincristine sulphate stock solution (0.1 mg–0.108 μmol) and 21 μL of the AOT stock solution (0.096 mg–0.216 μmol) were added to 1 mL of either unfunctionalised or ALD-grafted IL (vincristine sulphate/AOT molar ratio 1:2), and mixed via vortexing in order to form a vincristine–AOT hydrophobic ion pair (HIP) *in situ*, thus allowing the drugs to be loaded into the oily droplets.

##### IL Surface Grafting with ALD

IL grafting with ALD was carried out following a thiol–maleimide conjugation strategy [14]. ST-MBS was incorporated first into IL, while ALD was separately thiolated by reacting its primary amino group with 2-iminothiolane. Thiolated ALD was then reacted with the ST-MBS-loaded IL. The reactions were carried out in a 5:1 ALD/2-iminothiolane molar ratio, and in a 3:1 2-iminothiolane/ST-MBS molar ratio, allowing ST-MBS saturation.

Briefly, 1.2 mL of 0.28 mg/mL 2-iminothiolane solution (2.4 μmol) was reacted with 3.90 mg of sodium ALD (12 μmol) for 2 h at room temperature, under nitrogen steam in a glass vial (sealed with a rubber stopper), in order to minimise the oxidation of the thiol groups at the disulfide bridges. Separately, 1 mg of ST-MBS was pre-dissolved in 50 μL of DMSO; 20 µL of this solution (0.85 µmol) was added to 2 mL of IL under vortex stirring. Subsequently, the ALD/2-iminothiolane reaction mixture was added to ST-MBS-loaded IL and left to react for 2 h under magnetic stirring at room temperature. ALD-grafted IL was then purified by centrifugation; the mixture was centrifuged at 62,000× *g* (Allegra^®^ 64R centrifuge, Beckman Coulter, Palo Alto, CA, USA) for 5 min and the pellet was resuspended in 2 mL of water.

##### Fluorescence Labelling of Nanoemulsions

Nanoemulsions were labelled with three different fluorescent probes: 6-cou, NR and IR780-SDS. To this aim, stock solutions of the fluorescent probes were prepared in DMSO: 2 mg/mL for 6-cou and IR780-SDS, and 1 mg/mL for NR. These stock solutions were properly diluted in the nanoemulsions in order to obtain the following final concentrations: 0.1 mg/mL for 6-cou, and 0.05 mg/mL for NR and IR780-SDS. The content of the fluorescent probes was assessed using a Lambda 2 UV–vis spectrophotometer (Perkin Elmer, Waltham, MA, USA) after 20 µL of the labelled nanoemulsions was diluted in 1 mL methanol and centrifuged at 14,300× *g* (MPW55, Medical Instruments, San Lazzaro di Savena, Italy) for 5 min: the selected wavelengths were λ = 450 nm for 6-cou, λ = 550 mm for NR and λ = 705 nm for IR780-SDS.

#### 2.4.3. Formulation Characterisation

##### Nanoemulsion Particle Size and Zeta Potential

The dynamic light scattering technique (DLS; 90 Plus, Brookhaven, NY, USA) was used to determine the mean droplet size, polydispersity index (PDI) and Zeta potential of the IL-based formulations, at 25 °C and in triplicate. Measurement angles were 90° for particle size and 15° for Zeta potential.

The formulations under study underwent optical microscopy on a DM2500 microscope (Leica Microsystems, Wetzlar, Germany), coupled with a M480 camera (Motic, Barcelona, Spain).

##### Vincristine % Recovery and Entrapment Efficiency (EE%) in the Nanoemulsions

Vincristine % recovery, defined as the ratio between its actual and theoretical concentration in the nanoemulsion, was determined using high-pressure liquid chromatography (HPLC) after 50 μL of the nanoemulsion was diluted in 100 μL acetonitrile and centrifuged at 14,300× *g* (MPW55, Medical Instruments, San Lazzaro di Savena, Italy) for 5 min.

Vincristine EE%, defined as the ratio between its amount in the oily phase and the total in the nanoemulsion, was determined by separating the lipid pellet by centrifugation. Briefly, 500 μL of the formulation under study was centrifuged at 62,000× *g* (Allegra^®^ 64R centrifuge, Beckman Coulter, Palo Alto, CA, USA) for 15 min. The supernatant (containing the non-entrapped drug) was then removed, while the lipid pellet (containing the entrapped drug) was resuspended in 500 μL water. A total of 50 µL of the resuspended lipid pellet was then diluted with 100 µL of acetonitrile and centrifuged at 14,300× *g* (MPW55, Medical Instruments, San Lazzaro di Savena, Italy) for 5 min, prior to HPLC analysis.

##### Vincristine HPLC

Vincristine was analysed by HPLC via isocratic elution. The HPLC system was composed of a Jasco PU 1580 pump, a Jasco UV 1575 UV–visible detector (Jasco Corporation, Tokyo, Japan) and CromatoPlus software (Release 1.2.13) for data analysis (Aqualis srl, Nerviano, Italy). A 25 cm C18 Beckmann column was used. The mobile phase was phosphate buffer 0.02 M, pH 4.7—methanol 36:64. The flow rate was set to 1 mL/min. The Jasco UV–visible detector was set at λ = 276 nm. The retention time of vincristine was approximately 7 min.

##### Quantification of IL-Grafted ALD

Preliminary Ca^2+^ titration with ALD was carried out as follows. 500 µL of TRIS buffer (24.2 mg/mL–0.2 M, adjusted to pH = 10.0) and 50 µL of NET (1 mg/mL–2.17 mM) solution were placed into a glass test tube. The solution was blue due to the presence of the free NET indicator. Following the addition of 10 µL of CaBr_2_ (10 mg/mL–50 mM) solution and vortex mixing, the solution turned purple due to the formation of the NET-Ca^2+^ complex (Figure S1). ALD (0.65 mg/mL–2 mM) titrating solution was then added, 10 µL at a time; when complete, Ca^2+^ chelation by ALD occurred and the solution turned blue again. The ALD equivalents (ALD_eq_) of 50 mM CaBr_2_ were determined in this way.

ALD-IL conjugation efficiency was estimated in a similar way, via an ALD-titration method that compared unfunctionalised and ALD-grafted IL. Indeed, in the case of nanoemulsions, the contribution to the colour change by the phosphate groups of the lecithin (which can complex Ca^2+^) must be taken into account, in addition to the contribution from the grafted ALD. To this aim, in separate experiments, 200 µL of either unfunctionalised or ALD-grafted IL was added to the NET-Ca^2+^ complex, without obtaining a colour change. The titrating ALD solution was then added, 10 µL at a time, under vortexing, until the mixture turned blue (Figure S1). Therefore, the contribution of IL, either unfunctionalised or ALD-grafted, to the colour change was estimated from the ALD_eq_ difference between this experiment and the previous one, without nanoemulsions. Consequently, the ALD-IL conjugation efficiency was determined from the ALD_eq_ difference between ALD-grafted IL and unfunctionalised IL.

##### Affinity for Ca_3_(PO_4_)_2_ Column

The affinity of ALD-grafted IL for Ca^2+^, the main component of bone tissue, was verified by means of a Ca_3_(PO_4_)_2_ column, prepared as follows: 500 mg of Ca_3_(PO_4_)_2_ was suspended in 20 mL of water, and then poured into a syringe column. All the operations on the column were performed under nitrogen pressure (2 bars). The column was first packed, then conditioned with 2 mL of water. Subsequently, in separate experiments, 250 µL of unfunctionalised and ALD-grafted IL, both labelled with the fluorescent probe 6-cou (0.1 mg/mL), was diluted with 250 µL water and eluted with 1 mL of water, which was collected in a vial. Afterwards, to remove the labelled formulations bound to the column, 2 washing cycles, with 1 mL of ethanol each, were performed and collected in another vial.

The aqueous (unbound) and ethanol (bound) fractions that were collected were analysed on a Lambda 2 UV–vis spectrophotometer (Perkin Elmer, Waltham, MA, USA) at λ = 450 nm. The latter underwent centrifugation at 14,300× *g* (MPW55, Medical Instruments, San Lazzaro di Savena, Italy) for 5 min, before reading. The aqueous fraction was extracted with 1 mL of CHCl_3_, and the organic phase was then isolated and dried under nitrogen flow. The solid residue was dissolved in 1 mL of ethanol, and centrifuged at 14,300× *g* (MPW55, Medical Instruments, San Lazzaro di Savena, Italy) for 5 min before reading. The binding affinity of the formulations for the Ca_3_(PO_4_)_2_ column was expressed as the ratio between the 6-cou content in the ethanol fraction (bound) vs. the sum of the ethanol (bound) and aqueous (unbound) fractions.

#### 2.4.4. Cell Studies

##### Cytotoxicity Studies

The cytotoxicity of the vincristine-AOT loaded formulations was evaluated using an MTT assay, after 72 h treatment, in U2OS and HOS cells, according to a literature method [9,11].

##### Boyden Chamber Invasion Studies

The capability of the formulations under study to inhibit the invasion of U2OS and HOS cells was assessed using a Boyden chamber, following a literature method [9,11]. The effects of vincristine loading into IL and ALD grafting onto IL were both investigated. Prior to the assays with vincristine-based formulations, preliminary cytotoxicity experiments were performed after 6 h treatment with crystal violet staining in order to exclude early cytotoxicity, which can prevent the correct evaluation of migration inhibition [9,11].

#### 2.4.5. Studies on Isolated Tissues

Experiments were carried out on freshly excised mouse scapulae to evaluate the capability of unfunctionalised and ALD-grafted IL to accumulate in bone tissue *ex vivo*. 6-cou (0.1 mg/mL)-labelled formulations were employed. The scapulae were incubated separately in a 24-well plate with the formulations under study at 1, 2, 10 mg/mL 6-cou concentrations at 37 °C for 7 days. The scapulae were then washed and observed with fluorescence optical microscopy (DM5500 B, Leica, Wetzlar, Germany).

#### 2.4.6. Animal Studies

*In vivo* and *ex vivo* animal studies, using IVIS (IVIS Spectrum, PerkinElmer, Waltham, MA, USA) technology, were carried out to compare the biological fate of unfunctionalised and ALD-grafted IL after the injection of an established volume (100 μL) of labelled formulations into the tail veins of mice anesthetised with 3% isoflurane.

6-cou-labelled (0.1 mg/mL) and NR-labelled (0.05 mg/mL) formulations were used in preliminary experiments. The acquisitions for the 6-cou-labelled formulations were performed both at a fixed wavelength (λ_exc_ = 430 nm, λ_em_ = 560 nm) and using the “spectral unmixing” method (λ_exc_ = 430 nm, λ_em_ = 470), which allows the background noise to be minimised by changing the emission wavelength during the scan. Acquisitions with the NR-labelled formulations (λ_exc_ = 535 nm, λ_em_ = 580 nm) were performed in trans-luminescence mode.

Finally, IR780-SDS-labelled (0.05 mg/mL) formulations (acquisition at λ_exc_ = 710 nm, λ_em_ = 800 nm) were subjected to complete biodistribution experiments, with a special focus on the head and limbs.

#### 2.4.7. Statistical Analysis

Data are shown as mean ± SEM. Statistical analyses were performed with Prism 5.0 software (GraphPad Software, La Jolla, CA, USA) using one-way ANOVA and the Bonferroni test.

## 3. Results

Vincristine sulfate is a water-soluble salt that is not, per se, suitable for loading into the oily matrix of nanoemulsions. Therefore, in view of evidence in the literature [15], an *in situ* HIP method, performed at a 2:1 AOT/vincristine molar ratio, was used to enhance vincristine loading into IL. An acceptable EE% (44.5%), which increased to 66.0% in the case of ALD-grafted IL, was achieved using this method. It can be hypothesised that the negatively charged bisphosphonate, upon grafting to the oily IL droplets, electrostatically interacts with the basic groups of vincristine, which are protonated at physiological pH, thus improving drug EE%. ALD grafting to IL was achieved by means of a two-step reaction. In the first, the ALD amino group was reacted with 2-iminothiolane [14] to insert a thiol group in the molecule. This process was carried out under nitrogen steam in order to maintain mild reducing conditions, thus avoiding oxidation to disulfide [12]. The second step involved the reaction of thiolated ALD with IL, which had previously been loaded with the maleimide–lipid ST-MBS. No intermediate purification was performed between the two steps, and the oily ALD-grafted IL droplets were finally separated from the reaction mixture using an ultracentrifugation/resuspension procedure [12]. It is worth noting that excesses of ALD-to-2-iminothiolane and 2-iminothiolane-to-ST-MBS were used to improve thiolated ALD formation and ST-MBS saturation, respectively. A mean 0.88 μg ALD/mg lipid ratio was obtained, which corresponds to a conjugation efficacy of 63.7 ± 4.7%, calculated with reference to the μmol ST-MBS, which is the limiting reagent. Owing to its established clinical employment via the intravenous (i.v.) route, IL mean droplet size is around 250 nm with narrow polydispersity (<0.1), which indicates that a single-size population is present. In our work, neither vincristine–AOT loading nor ALD grafting had a considerable impact on IL droplet size. However, polydispersity did increase and the absolute Zeta potential values were lower, indicating a partial alteration of the nanoemulsions. It is important to note that similar increases in polydispersity may reflect the presence of droplets in the micrometre range, which might induce emboli formation. This possibility was excluded via observation under optical microscopy (Figure S2). The physico-chemical characterisation of the nanoemulsions is shown in Table 1.

**Figure 1:**
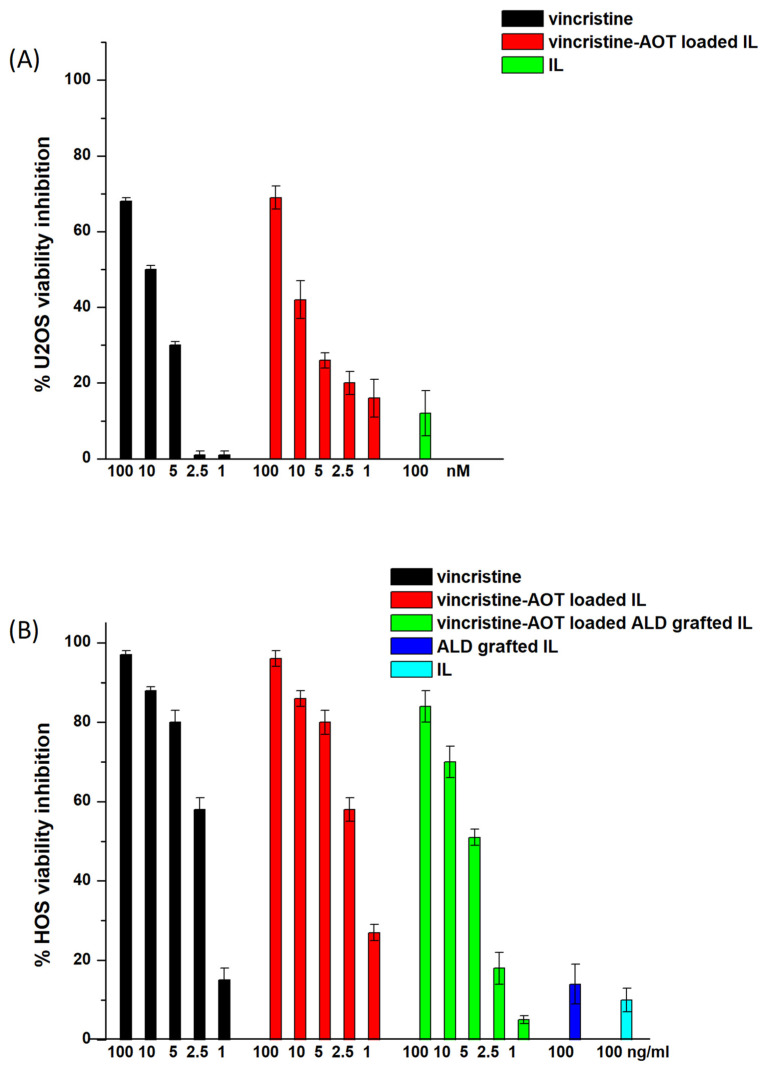
3-(4,5-dimethyl thiazol-2-yl)-2,5-diphenyltetrazolium bromide (MTT) assay on U2OS and HOS cells after 72 h treatment with vincristine-loaded formulations. (**A**) U2OS (n = 3); (**B**) HOS (n = 5). Abbreviations: ALD: alendronate sodium; AOT: sodium docusate; IL: Intralipid^®^ 10%.

Vincristine–AOT-loaded IL was then tested for cytotoxicity towards U2OS and HOS osteosarcoma cells by means of a MTT assay after 72 h of treatment (Figure 1). No significant differences were noted between the IL-loaded and free drugs along the entirety of the dose–response curve. Moreover, vincristine–AOT-loaded and ALD-grafted IL was also tested in a selected series of experiments on HOS cells. However, no increase in cytotoxicity was observed with ALD grafting, and no further MTT assays were therefore carried out with functionalised IL. Indeed, although nitrogen-containing bisphosphonates have been shown to possess cytotoxic activity [16,17], this can only be observed after long treatment times (i.e., 1 week) [18] which are unsuitable for the evaluation of conventional cytotoxic drugs. Moreover, while zoledronate exerts a certain dose-dependent cytotoxic effect, ALD only exerts its cytotoxicity at very high doses [19,20], which are not compatible with the amounts conjugated onto the IL surface.

**Figure 2:**
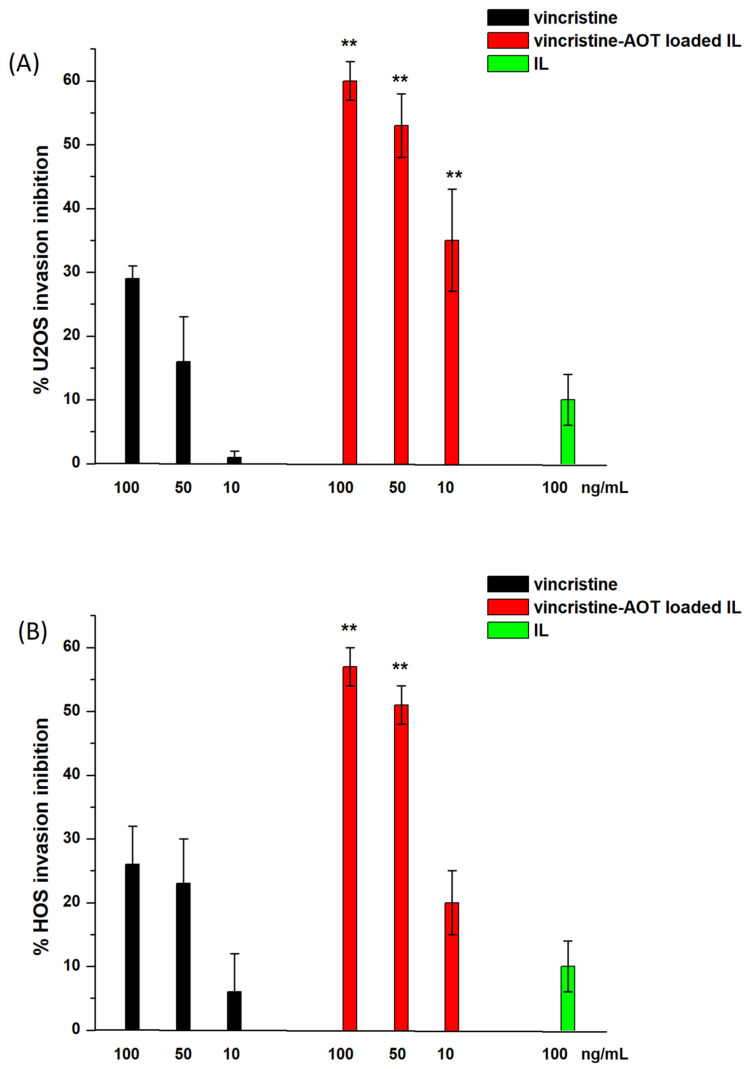
Invasion assay on U2OS and HOS cells with vincristine-loaded formulations (n = 5). (**A**) U2OS; (**B**) HOS. Abbreviations: AOT: sodium docusate; IL: Intralipid^®^ 10%. Statistical analysis: ** *p* < 0.01 vincristine–AOT-loaded IL vs. vincristine.

Nonetheless, ALD is able to inhibit cell invasion, a relevant step in the metastatisation process, and, although this effect normally occurs at a high dose [20], conjugation to the oily IL droplets may significantly modify the therapeutic dose. With that in mind, the invasion-inhibitory effect of ALD should be evaluated together with that of vincristine, which is based on microtubule inhibition [21,22,23]. Therefore, prior to investigating the effect of ALD, dose–response was established for the invasion inhibition of vincristine-loaded IL on U2OS and HOS cells (Figure 2). This was obtained by means of invasion experiments using a Boyden chamber after 6 h of treatment. Given that vincristine exerts potent cytotoxic activity, preliminary crystal violet experiments were performed in order to exclude any cytotoxic effect during this timeframe with the selected drug doses (Figure S3), as this would hamper the correct evaluation of invasion inhibition.

**Figure 3:**
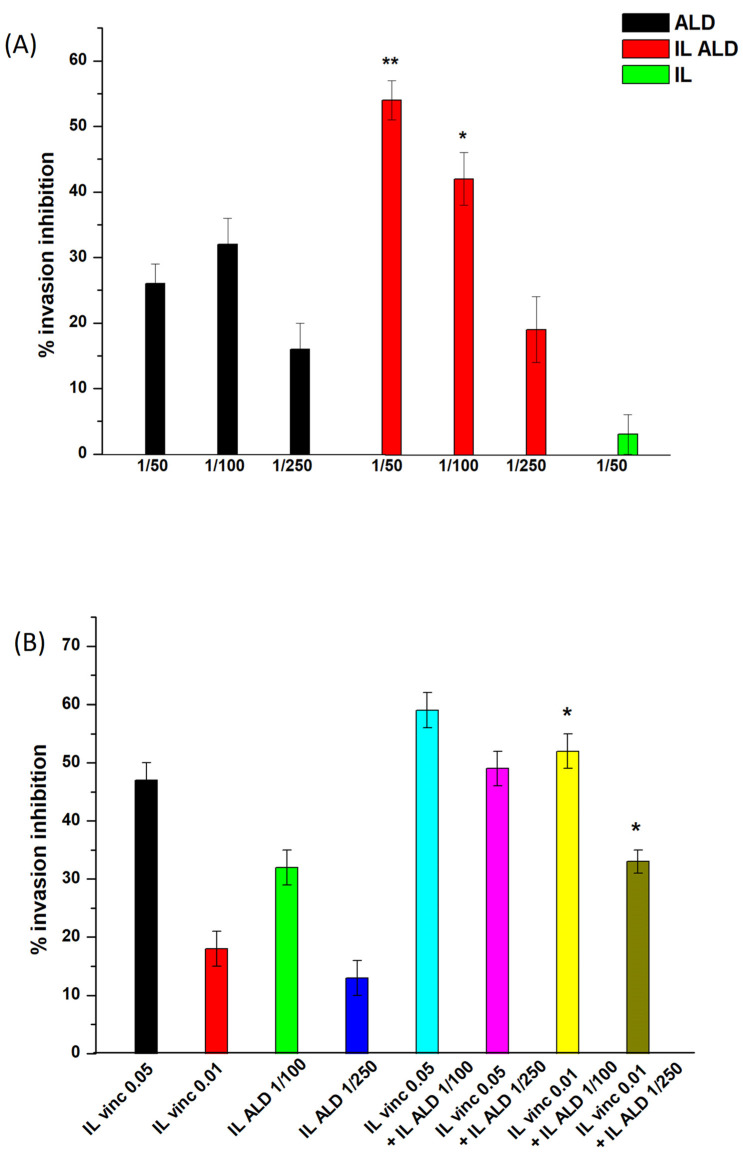
Invasion assay on U2OS cells with ALD-grafted and vincristine–AOT-loaded nanoemulsions (n = 5). (**A**) Effect of ALD grafting; (**B**) effect of ALD grafting and vincristine loading. Abbreviations: ALD: alendronate sodium; IL: Intralipid^®^ 10%; IL ALD: ALD-grafted IL; vinc: vincristine. Statistical analysis: (**A**) * *p* < 0.05 ALD-grafted IL vs. free ALD, ** *p* < 0.01 ALD-grafted IL vs. free ALD; (**B**) * *p* < 0.05 ALD-grafted and vincristine–AOT-loaded IL vs. vincristine-AOT-loaded IL and vs. ALD-grafted IL.

As can be seen in Figure 2, loading into IL caused a significant increase in the invasion inhibition capacity of vincristine in both cell lines, while blank IL exerted no effect. This paved the way for an evaluation of the synergistic effect of ALD grafting and vincristine loading in the same vehicle (Figure 3). U2OS cells were selected for this set of experiments.

As shown in Figure 3, the effect of ALD grafting alone was first assessed in a dose-dependent manner. Interestingly, while free ALD exerted a negligible effect, as is in accordance with the literature [20], IL-grafted ALD inhibited cell invasion in a dose-dependent manner, with significant differences with respect to free ALD. However, based upon the amounts of ALD grafted and vincristine loaded into IL, the IL doses needed to cause invasion inhibition were at least 20-fold higher for ALD. Therefore, the synergism between ALD and vincristine was investigated by mixing different amounts of ALD-grafted IL and vincristine–AOT-loaded IL in different ratios. Importantly, an additive effect was observed using the two therapeutic agents, with significant differences with respect to the single agents being noted, in the case of low-dose vincristine.

Based upon these promising results, further studies were carried out, using fluorescently labelled formulations, in order to assess the bone-targeting ability of the ALD-grafted formulations. Firstly, the affinity of the 6-cou-labelled nanoemulsions for a Ca_3_(PO_4_)_2_ column was assessed. As reported in Table 2, % binding affinity increased in the case of ALD-grafted IL, regardless of vincristine–AOT loading.

In the next step, mouse scapulae were incubated with 6-cou-labelled IL, either unfunctionalised or ALD-grafted, at 37 °C for 7 days, then washed and observed with fluorescence optical microscopy. As can be noticed qualitatively in Figure S4, ALD grafting enhanced the fluorescence associated with the scapulae.

Finally, *in vivo* studies using fluorescently labelled nanoemulsions were performed on healthy mice by means of IVIS technology in order to evaluate the influence of ALD grafting on IL biodistribution, with a particular focus on the bones. Due to the formulation’s biocompatibility and homogeneous droplet size, the animals showed no signs of systemic toxicity after the i.v. administration of the labelled formulations, regardless of whether they were unfunctionalised or ALD-grafted.

**Figure 4:**
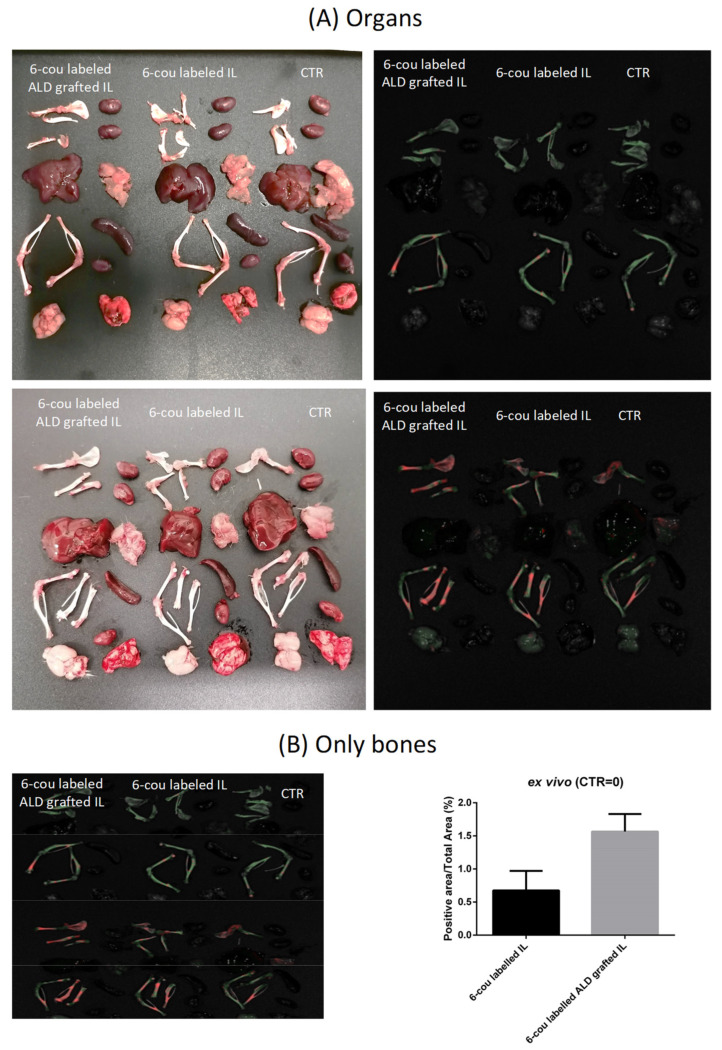
*Ex vivo* biodistribution using IVIS (spectral unmixing mode), 48 h after administration of 6-cou labelled nanoemulsions (n = 2). (**A**) Organ acquisition; (**B**) bones only, acquisition and quantification. Abbreviations: 6-cou: 6-coumarin; ALD: alendronate sodium; CTR: control; IL: Intralipid^®^ 10%.

In preliminary experiments, nanoemulsions were fluorescently labelled with either 6-cou or NR, and a time-dependent fluorescence decay for up to 48 h after administration was detected (Figure S5) using 6-cou labelled formulations and “spectral unmixing” acquisition mode (to minimise background noise). After animal sacrifice, negligible fluorescence was observed in the tissues, compared to the bones, where a large signal was observed in the animals treated with ALD-grafted formulations (Figure 4).

**Figure 5:**
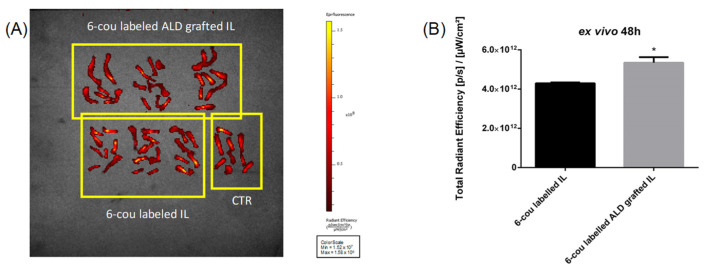
*Ex vivo* quantification of mouse-bone accumulation using IVIS, 48 h after administration of 6-cou-labelled nanoemulsions (n = 3). (**A**) Fixed-wavelength acquisition; (**B**) quantification. Abbreviations: 6-cou: 6-coumarin; ALD: alendronate sodium; CTR: control; IL: Intralipid^®^ 10%. Statistical analysis: * *p* < 0.05 ALD-grafted IL vs. unfunctionalised IL.

Moreover, *ex vivo* experiments performed using fixed-wavelength acquisition confirmed that ALD grafting is capable of improving the bone accumulation of 6-cou-labelled IL (Figure 5). This can also be qualitatively appreciated using *in vivo* trans-luminescence acquisition after the administration of the NR-labelled formulation; in the case of ALD-grafted IL, fluorescence was specifically detected in the mouse legs (Figure S6).

**Figure 6:**
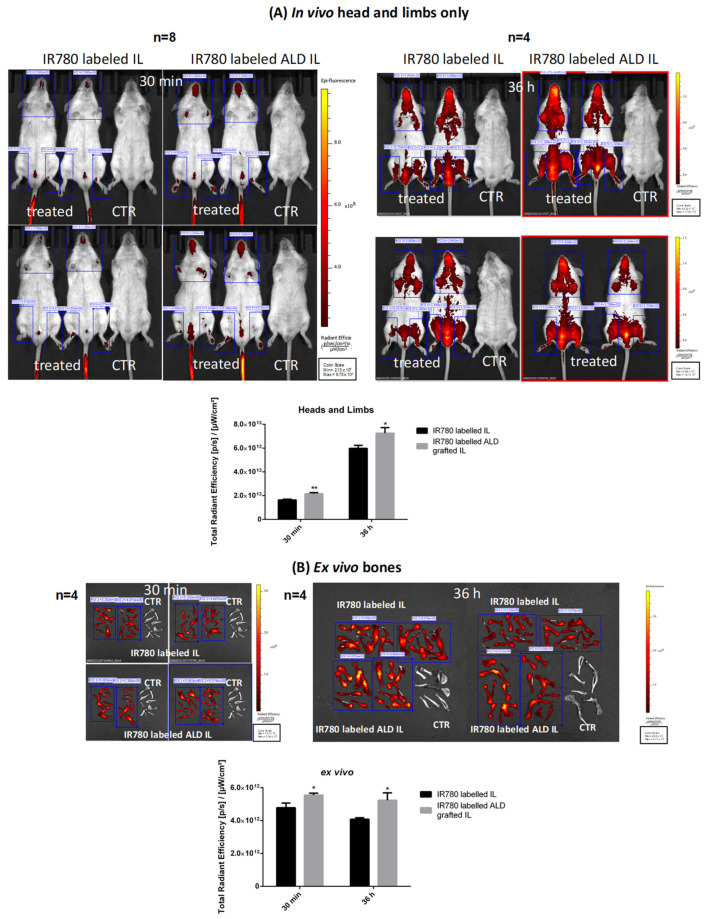
*In vivo* and *ex vivo* quantification of mouse-bone accumulation after administration of IR780-SDS-labelled nanoemulsions. (**A**) *In vivo* head and limbs only, acquisition and quantification; (**B**) *ex vivo* bone acquisitions and quantification. Number of replicates (n) indicated in the figure. Abbreviations: ALD: alendronate sodium; ALD IL: ALD-grafted IL; CTR: control; IL: Intralipid^®^ 10%; IR780: 2-[2-[2-Chloro-3-[(1,3-dihydro-3,3-dimethyl-1-propyl-2H-indol-2-ylidene)ethylidene]-1-cyclohexen-1-yl]ethenyl]-3,3-dimethyl-1-propylindolium. Statistical analysis: * *p* < 0.05 ALD-grafted IL vs. unfunctionalised IL; ** *p* < 0.005 ALD-grafted IL vs. unfunctionalised IL.

Given that preliminary results with 6-cou and NR showed that all labelling led to the detection of the fluorescent dye in the bone, but with high background noise, other experiments were performed with IR780 in order to decrease this background interference (Figure S7). However, being a cationic dye, it interacts with the negatively charged surface of IL, leading to droplet enlargement, which prevents its usage in animal experiments. The effect of this positive charge was quenched by substituting the iodide counter-ion with SDS, thus forming a more lipophilic IR780-SDS ion pair, which can be then loaded into IL without affecting droplet size. As shown in Figure 6, the IR780 signal is clearly visible, allowing us to appreciate how the administered nanoemulsions are predominantly biodistributed in the head and limbs. Moreover, *in vivo* quantification highlighted that ALD-grafted and unfunctionalised IL showed significant differences in head and limb accumulation even 30 min after administration. These differences were maintained 36 h after administration. *Ex vivo* experiments, obtained after the sacrifice of selected animals from the two cohorts at different timeframes after administration (30 min and 36 h, respectively), show that the accumulation of ALD-grafted IL in the bones was considerably higher than that of the unfunctionalised agent.

## 4. Discussion

Compared to alternative nanocarriers, injectable lipid nanoemulsions can claim a great many advantages, including biocompatibility, physico-chemical stability, easy sterilisation (by steam and/or filtration) and high scalability for large batch-based production [7]. In particular, they allow chemical surface reactions to be freely performed without altering droplet size [12]. The maleimide–thiol reaction was selected for ALD grafting in this case because of its chemical selectivity [24]. Indeed, the maleimide moiety reacts selectively with a free thiol group that is chemically inserted onto the ALD molecule [14]. Free thiol groups are absent from all components of the lipid matrix, as well as from most of the potential drug candidates to be loaded into IL.

Bisphosphonate functionalisation has already been exploited for bone targeting by nanocarriers [25,26], and the conjugation of bisphosphonates with lipid moieties has shown promising results in animal models [27]. However, from a pharmacological standpoint, the idea of merging the antitumour activities of bisphosphonates and conventional cytotoxic drugs within the same nanoemulsion carrier is innovative, but is hampered by several concerns, with the main one being the therapeutic dose. Indeed, nanocarriers can generally either be loaded or grafted with low amounts of selected compounds, meaning that only potent drugs can be successfully delivered using nanocarriers. While vincristine is a microtubule inhibitor, which can act on several tumour-growth mechanisms (i.e., proliferation, migration) at low doses [4], only nitrogen-containing bisphosphonates exert any antitumour activity [17]. ALD, which is a nitrogen-containing bisphosphonate, was selected because it can be chemically grafted onto nanoemulsion droplets [14]. However, its cytotoxic activity can only be achieved at very high doses and after long exposure [20]. However, bisphosphonates may also prevent bone metastases by inhibiting osteoclast activity and, thus, counteract tumour osteolytic activity, which is important in permitting tumour grafting in bone tissue [28].

In our setting, the low dose of ALD that is grafted onto IL is not sufficient to exert a cytotoxic effect, as assessed by the MTT assay. Nonetheless, ALD is capable of inhibiting cell invasion, which is an important step in the metastatisation process, and displays an additive effect with vincristine. Despite the high dose dependence of this activity [20], it was possible to estimate it in an *in vitro* experiment with the Boyden chamber using suitable vincristine–ALD ratios.

It is worth noting that, besides activity towards osteosarcoma cells, the principal role ascribed to ALD grafting onto the IL surface, in our approach, is the bone targeting of IL-loaded drugs, such as vincristine, in order to prevent/counteract bone metastasis formation in a relevant number of malignancies. The anti-metastatic effect of these nanoemulsions would be multifaceted. On the one hand, vincristine would inhibit both tumour-cell invasion into the bone and subsequent proliferation. On the other hand, ALD would not only increase the delivery of vincristine to the bone, but also increase its inhibitory activity against tumour-cell invasion and counteract tumour-cell grafting by inhibiting the osteolytic activity of osteoclasts.

Suitable *ex vivo/in vivo* models are needed to assess the potential bone-targeting ability of ALD-grafted IL. In fact, IVIS technology makes use of fluorescently labelled formulations and, thus, allows both *in vivo* real-time studies and *ex vivo* evaluations to be performed after animal sacrifice. In this way, a plethora of data (pharmacokinetic, biodistribution) can be obtained from the same live animal, minimising the number of subjects for each cohort and allowing the time of sacrifice in *ex vivo* evaluations to be optimised [29]. Therefore, encouraging preliminary data for the assessment of the potential of IL, and in particular of ALD-grafted IL, to target the bone have been obtained in this experimental study thanks to IVIS technology. While the use of IVIS in this field is not new [25,26,27,30], our approach has allowed us to appreciate the versatility of the IVIS technology in evaluating bone targeting by employing different fluorescent probes and acquisition modes.

Bone tissue is a common metastatic site for circulating tumour cells because the blood flow in the bone marrow is relatively slow, and adhesion receptors are expressed on bone marrow capillary endothelial cells that support the localisation of cancer cells. When tumour cells enter the sinusoidal microvessels of the bone marrow and colonise the bone, they form niches that facilitate tumour growth by involving complex crosstalk between tumour cells and the bone microenvironment. Indeed, tumour cells secrete various cytokines, such as interleukin-6, which can increase the expression of the receptor activator of nuclear factor kappa-B ligand (RANKL) in osteoblasts, and activate osteoclasts to increase bone resorption by secreting parathyroid hormone-related peptide. In addition, immune cells, stromal cells, fibroblasts and endothelial cells form a bone-metastasis microenvironment similar to an inflammation site [31].

A vicious cycle is therefore formed between tumour growth and bone resorption. Osteolytic metastases, most frequent in the case of breast cancer, are a consequence of increased bone resorption due to tumour cells [32]. Osteoblastic bone metastases occur mostly in prostate cancer [33]. In this case, together with an increase in osteoclastogenesis, osteoblasts are also activated by prostate cancer cells and the tumour microenvironment, leading to the accumulation of newly formed bone. Despite major efforts in targeting this vicious cycle, success has so far been limited, with the exception of bone-targeted therapies, such as bisphosphonates and denosumab (anti-RANKL monoclonal antibody), meaning that the complexity of the system somehow impairs the effective role of antitumour agents [34]. Interestingly, adjuvant bisphosphonates have been demonstrated to reduce the risk of bone metastases in patients with iatrogenic or physiological menopause, compared to currently approved bone targeting agents, while data on denosumab are contradictory and require further investigation [32].

Several strategies have been exploited for the targeting of drug-loaded nanocarriers to bone metastases, and have been designed specifically for the features of such a specific microenvironment. These strategies include bio-mimetic coating and immune therapy. However, treatments that are based on agents that regulate osteoclastic activity are the most widespread [31]. In particular, aminobisphosphonates such as ALD and zoledronate, thanks to their aforementioned claimed superiority over denosumab, have been successfully used as targeting ligands for bone metastases. ALD is a popular ligand because it has a primary amine, which allows for easy conjugation with drug carriers through various chemical linkers [35].

Although ALD-mediated targeting itself cannot guarantee the selective targeting of bone metastases with respect to healthy bone, it should be pointed out that each step in the metastatic process is, conceivably, a drug target in preventing metastasis; in the case of bone metastasis, this would mean preventing the epithelial–mesenchymal transition associated with the initial invasion and/or modulation of the pre-metastatic niche, which recruits tumour cells from distant tumour sites, in the surrounding healthy bone. In this context, the existence of the enhanced permeability and retention (EPR) effect [36] in metastasis is debatable, unlike in the primary tumour, with studies reporting that metastases exhibit the phenomenon, while others report otherwise, especially in early-stage metastasis. The passive targeting of nanocarriers to bone metastases might take advantage of the fenestrations in bone marrow capillaries [37], yet bone marrow accumulation is associated with the well-known haematological toxicities of chemotherapeutics-loaded nanocarriers [38]. However, in early-stage metastases, the passively targeted nanocarriers localised within the bone marrow are not associated with the bone [37]. On the contrary, in bone tumour models that had been implanted into the tibia of mice, ALD-coated nanoparticles, despite being mostly close to bone surfaces, were also partially taken up by bone marrow cells. Therefore, ALD-targeted nanoparticles may be a useful carrier system for chemotherapeutics that can prevent the growth and progression of bone-residing tumours [39], while reducing the myelosuppression associated with conventional nanoparticles.

Moreover, ALD-mediated targeting allows simultaneous pharmacological activity to be exerted against bone metastases, which might be attributable to three main modes of action due to the bisphosphonate itself: First, antiresorptive activity in the presence of clinically overt osteolytic bone metastases. Second, direct effects on tumour cells, not only via the suppression of local disease progression in the bone, but also via antitumour effects outside the bone. Even if this is probably an indirect effect, mainly due to osteoclastic bone resorption, direct effects on tumour cells, including impaired proliferation, migration, invasion, the induction of apoptosis and the inhibition of tumour-induced neoangiogenesis have been demonstrated. Third, aminobisphosphonates might have immuno-modulatory effects, in particular on macrophages and γδT cells, which are an antitumour subset of T cells. Accordingly, the rapid expansion of γδT cells, with secondary cytokine release, is responsible for the acute-phase reaction that occurs in up to 20% of treated patients, but has also been linked to cytotoxicity against myeloma cells, and has improved outcomes in patients with advanced-stage breast and prostate cancer [40].

## 5. Conclusions

In this experimental work, IL was loaded with vincristine and grafted with ALD, allowing the anticancer activity of the two compounds to be merged into one biocompatible nanocarrier, as demonstrated in cell studies, while also enhancing bone-targeted nanocarrier delivery, as assessed in preliminary animal studies with fluorescently labelled nanoemulsions using the IVIS technology. These encouraging results will drive further investigations into the pharmacological treatment of bone metastases, using suitable animal models of the disease.

## Figures and Tables

**Table 1:** Physico-chemical characterisation of nanoemulsions. Abbreviations: ALD: alendronate sodium; AOT: sodium docusate; EE: entrapment efficiency; IL: Intralipid^®^ 10%.

	Mean Size (nm)	Polydispersity	Z Potential (mV)	Grafted ALD (μg/mg Lipid)	Drug % Recovery	Drug EE %
Blank IL	268.4 ± 5.0	0.071	−38.49 ± 11.16	-	-	-
ALD-grafted IL	263.1 ± 4.5	0.158	−29.15 ± 2.16	0.88 ± 0.06	-	-
Vincristine–AOT-loaded IL	264.4 ± 3.3	0.173	−25.00 ± 5.17	-	103.5 ± 9.6	44.5 ± 3.0
Vincristine–AOT-loaded ALD-grafted IL	249.9 ± 3.5	0.194	−24.64 ± 11.53	0.88 ± 0.06	81.0 ± 12.3	66.0 ± 2.8

**Table 2:** Binding affinity of 6-cou-labelled nanoemulsions to the Ca_3_(PO_4_)_2_ column. Abbreviations: 6-cou: 6-coumarinM ALD: alendronate sodium; AOT: sodium docusate; IL: Intralipid^®^ 10%.

	Unfunctionalised IL	ALD-Grafted IL	Vincristine–AOT-Loaded ALD-Grafted IL
% binding affinity	52.5 ± 4.6	94.3 ± 3.8	86.0 ± 5.0

## Data Availability

The data presented in this study are available on request from the corresponding author.

## Supplementary Materials

The following supporting information can be downloaded at: https://www.mdpi.com/article/10.3390/biom14020238/s1. Figure S1. Colour change of NET in the presence (left) and in the absence (right) of Ca^2+^, in solution (A) and in IL (B). Abbreviations: IL: Intralipid^®^ 10%; NET: eriochrome black T. Figure S2. Optical microscopy of engineered formulations. (A) blank IL; (B) ALD-grafted IL; (C) vincristine–AOT-loaded IL; (D) ALD-grafted vincristine–AOT-loaded IL. Abbreviations: ALD: alendronate; AOT: sodium docusate; IL: Intralipid^®^ 10%. Figure S3. Crystal violet assay on U2OS and HOS cells after 6 h of treatment with vincristine-based formulations. Abbreviations: AOT: sodium docusate; IL: Intralipid^®^ 10%. (A) U2OS; (B) HOS. Figure S4. Fluorescence optical microscopy of mouse scapulae after incubation with different amounts of 6-cou-labelled nanoemulsions. Upper panel: 1 μg/mL 6-cou; 2 μg/mL 6-cou; 10 μg/mL 6-cou. Abbreviations: 6-cou: 6-coumarin; ALD: alendronate sodium; IL: Intralipid^®^ 10%. Figure S5. *In vivo* pharmacokinetics using IVIS (spectral unmixing mode) after administration of 6-cou-labelled nanoemulsions. (A) acquisitions; (B) quantification. Abbreviations: 6-cou: 6-coumarin; ALD: alendronate sodium; CTR: control; IL: Intralipid^®^ 10%. Figure S6. *In vivo* biodistribution in mouse legs using IVIS (trans-luminescence mode), 24 h after administration of NR-labelled nanoemulsions. Abbreviations: ALD: alendronate sodium; CTR: control; IL: Intralipid^®^ 10%; NR: Nile Red. Figure S7. *In vivo* biodistribution (total body) of IR780-SDS-labelled nanoemulsions, 30 min after administration. Abbreviations: ALD: alendronate sodium; CTR: control; IL: Intralipid^®^ 10%; IR780: 2-[2-[2-Chloro-3-[(1,3-dihydro-3,3-dimethyl-1-propyl-2H-indol-2-ylidene)ethylidene]-1-cyclohexen-1-yl]ethenyl]-3,3-dimethyl-1-propylindolium; SDS: sodium dodecyl sulfate.
