# Supplementary material for: Alendronate-Grafted Nanoemulsions for Bone-Targeted Vincristine Delivery: Preliminary Studies on Cell and Animal Models
Source: Biomolecules. 2024 Feb 18;14(2):238. doi: 10.3390/biom14020238 (PMC10886946; doi:10.3390/biom14020238)
Supplement: Supplementary file 1 [file biomolecules-14-00238-s001.zip › biomolecules-2830515-supplementary.pdf]

Alendronate-Grafted Nanoemulsions for Bone-Targeted Vincristine Delivery: Preliminary Studies on Cell and Animal Models

Ian Stoppa, Chiara Dianzani, Nausicaa Clemente, Annalisa Bozza, Valentina Bordano, Sara Garelli, Luigi Cangemi, Umberto Dianzani and Luigi Battaglia

Supplementary materials

Figure S1 Page 2

Figure S2 Page 3

Figure S3 Page 4

Figure S4 Page 5

Figure S5 Page 6

Figure S6 Page 7

Figure S7 Page 8

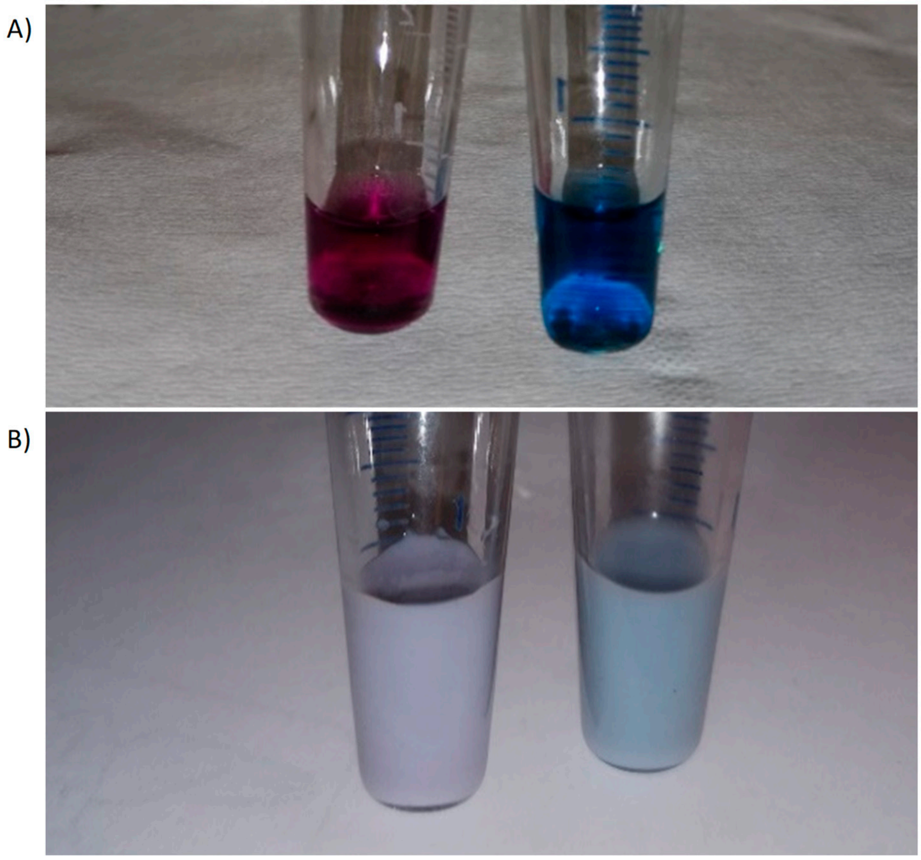

Figure S1. Colour change of NET, in the presence (left) and in the absence (right) of Ca^{2+}, in solution (A) and in IL (B). Abbreviations: IL: Intralipid® 10%; NET: eriochrome black T.

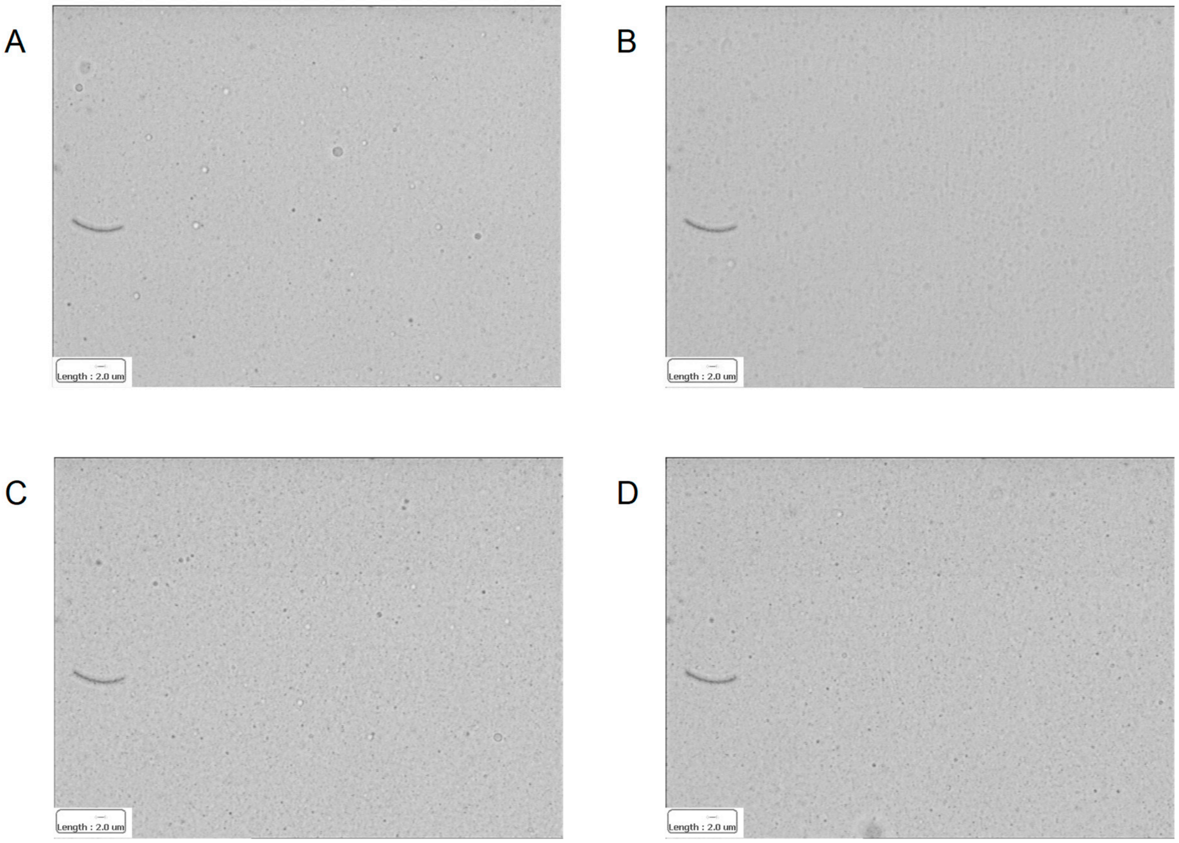

Figure S2. Optical microscopy of engineered formulations. A) blank IL; B) ALD-grafted IL; C) vincristine-AOT loaded IL; D) ALD-grafted vincristine-AOT loaded IL. Abbreviations: ALD: alendronate; AOT: sodium docusate; IL: Intralipid® 10%.

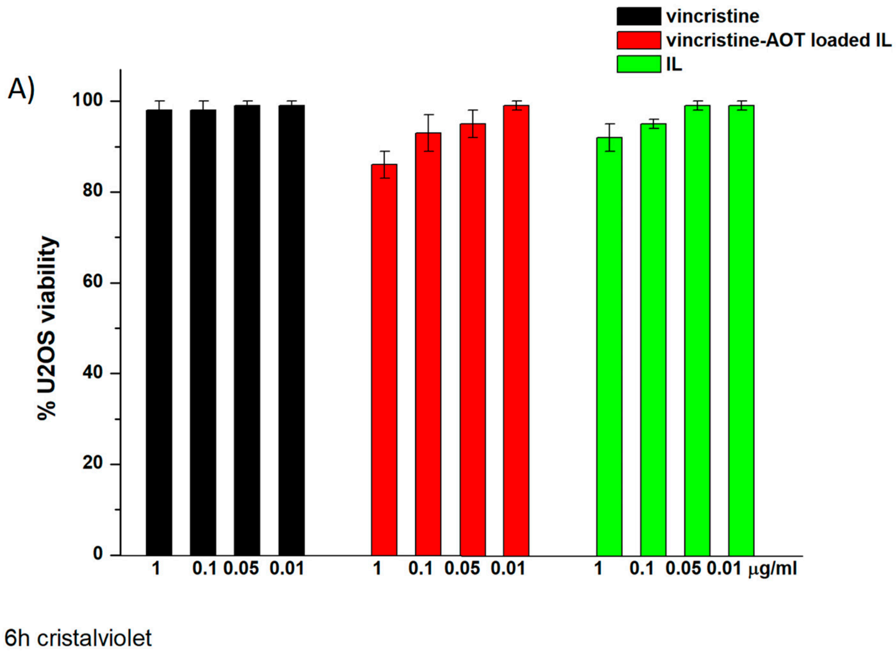

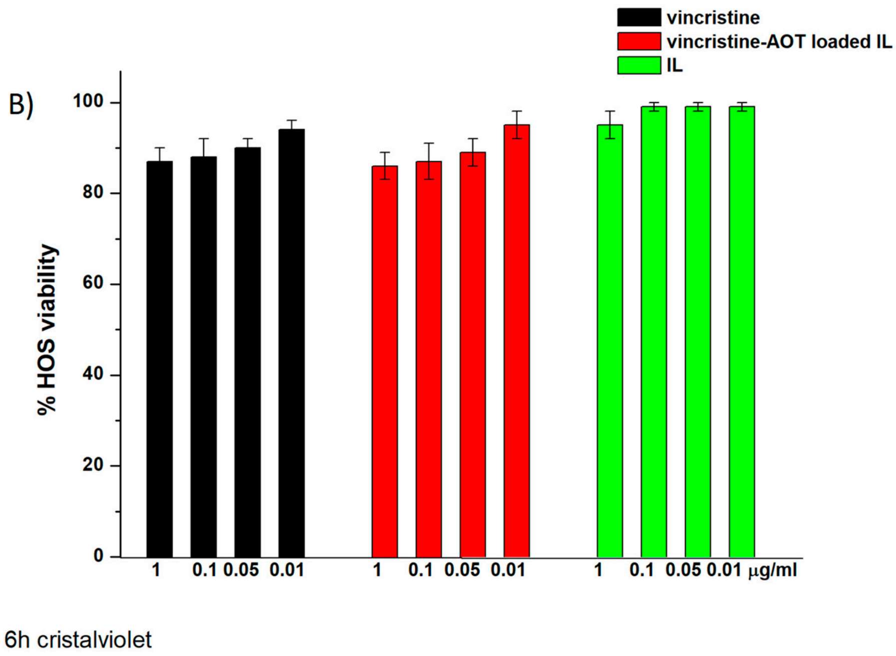

Figure S3. Crystal violet assay on U2OS and HOS cells after 6 hours of treatment with vincristine-based formulations. Abbreviations: AOT: sodium docusate; IL: Intralipid® 10%. A) U2OS; B) HOS.

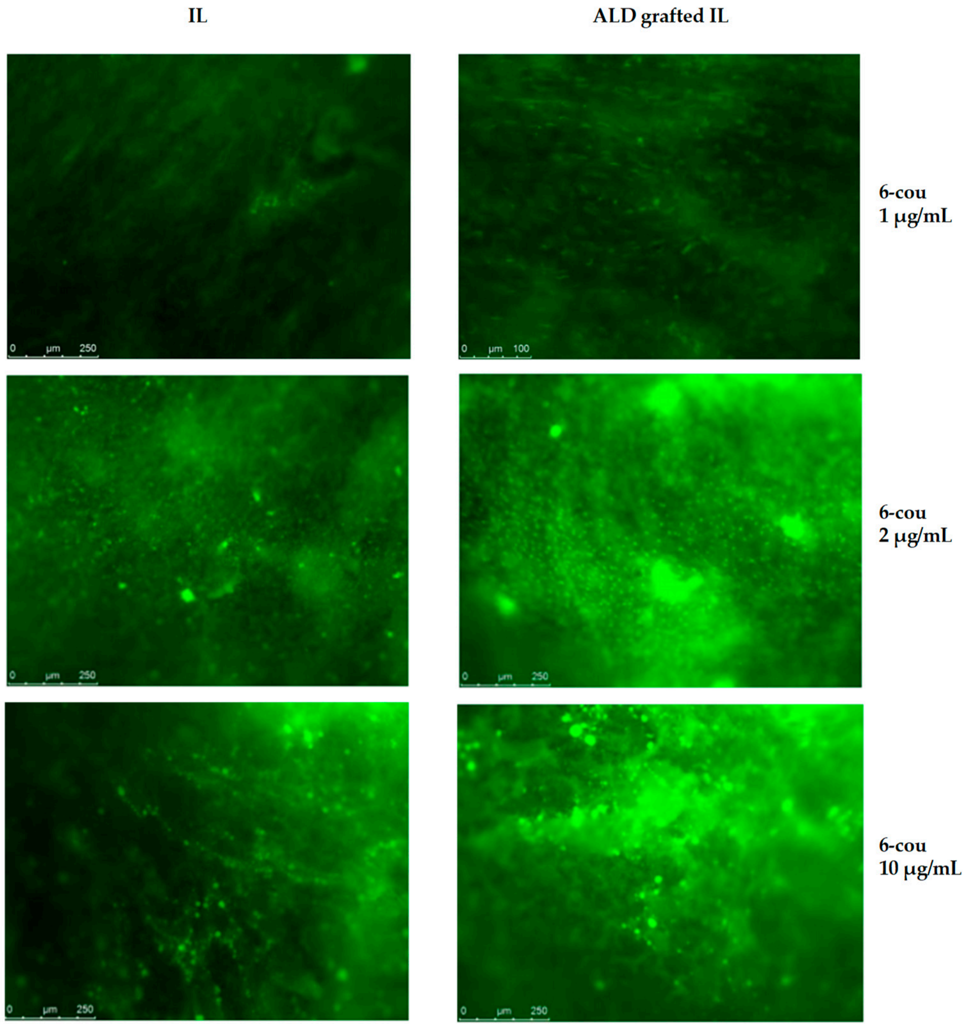

Figure S4. Fluorescence optical microscopy of mouse scapulae after incubation with different amounts of 6-cou-labelled nanoemulsions. Left panel: 6-cou labelled IL; right panel: 6-cou labelled ALD grafted IL. Upper panel: 1 µg/mL 6-cou; 2 µg/mL 6-cou; 10 µg/mL 6-cou. Abbreviations: 6-cou: 6-coumarin; ALD: alendronate sodium; IL: Intralipid® 10%.

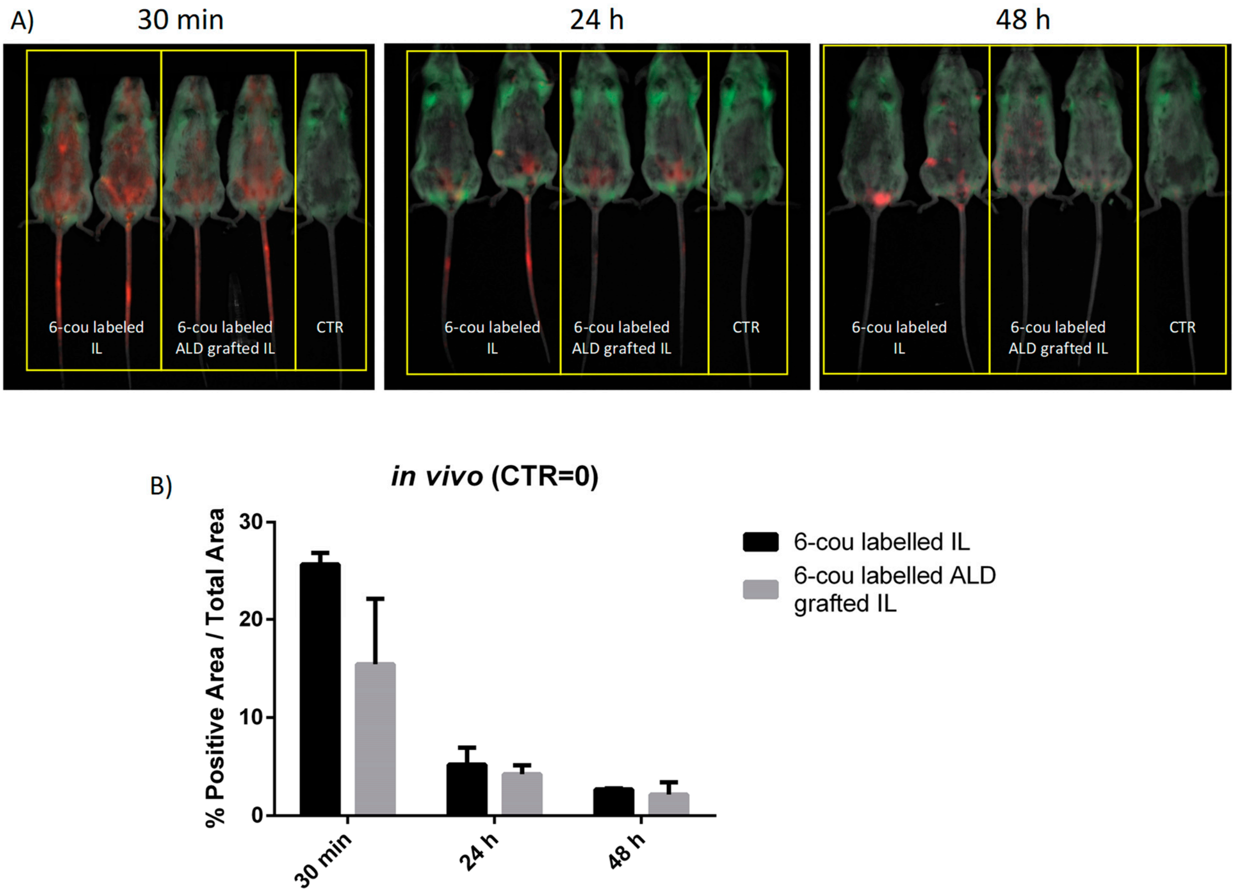

Figure S5. *In-vivo* pharmacokinetics using IVIS (spectral unmixing mode), after administration of 6-cou-labelled nanoemulsions (n=2). A) acquisitions; B) quantification. Abbreviations: 6-cou: 6-coumarin; ALD: alendronate sodium; CTR: control; IL: Intralipid® 10%.

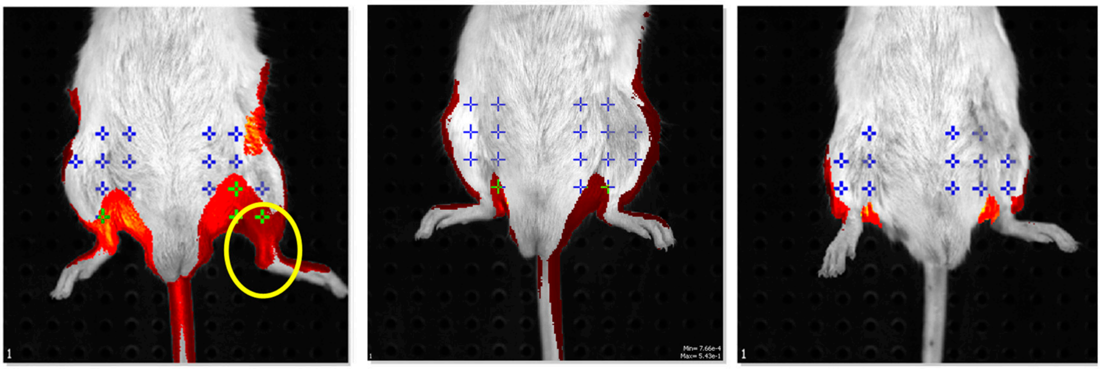

NR labeled
ALD grafted IL

NR labeled IL

CTR

Figure S6. *In-vivo* biodistribution in mouse legs using IVIS (trans-luminescence mode), 24 hours after administration of NR-labelled nanoemulsions. Abbreviations: ALD: alendronate sodium; CTR: control; IL: Intralipid® 10%; NR: Nile Red.

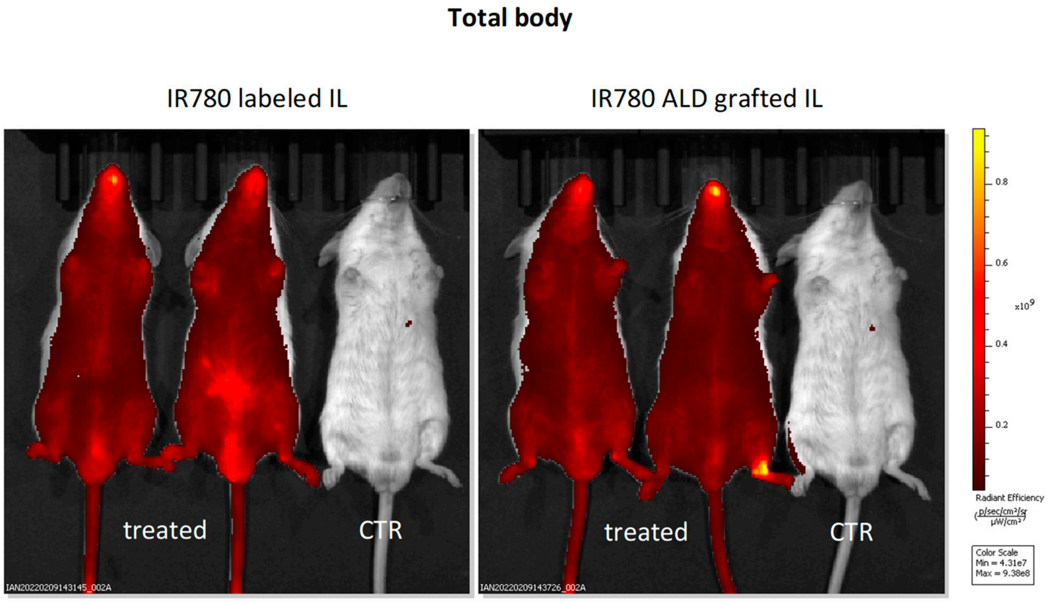

Figure S7. *In-vivo* biodistribution (total body) of IR780-SDS-labelled nanoemulsions, 30 minutes after administration. Abbreviations: ALD: alendronate sodium; CTR: control; IL: Intralipid® 10%; IR780: 2-[2-[2-Chloro-3-[(1,3-dihydro-3,3-dimethyl-1-propyl-2H-indol-2-ylidene)ethylidene]-1-cyclohexen-1-yl]ethenyl]-3,3-dimethyl-1-propylindolium; SDS: sodium dodecyl sulfate.
